# Association between pro-inflammatory proteins and neurofilament in plasma from persons with epilepsy

**DOI:** 10.1186/s12916-025-04425-z

**Published:** 2025-10-13

**Authors:** Mayuresh Sarangdhar, Sarah Akel, Saman Hosseini Ashtiani, Markus Axelsson, Johan Zelano

**Affiliations:** 1 Department of Clinical Neuroscience, Institute of Neuroscience and Physiology, Sahlgrenska Academy, University of Gothenburg, Gothenburg, Sweden; 2 Wallenberg Center of Molecular and Translational Medicine, Gothenburg University, Gothenburg, Sweden; 3 Department of Neurology, Member of ERN Epicare, Sahlgrenska University Hospital, Gothenburg, Sweden

**Keywords:** Drug-resistant epilepsy, Inflammation, Neurodegeneration, Biomarker, Plasma proteomics, Seizures

## Abstract

**Background:**

Inflammation and neurodegeneration are emerging as pathophysiological processes of interest in epilepsy. Seizures can both arise from and induce inflammation and difficult-to-treat epilepsy is linked to brain atrophy. However, the interplay between inflammation and neurodegeneration remains poorly understood in epilepsy. This study investigates the association between inflammatory proteins and plasma neurofilament light chain (NEFL or NfL), a known marker of neurodegeneration, particularly in relation to active epilepsy.

**Methods:**

We performed Olink proteomics on plasma from 176 epilepsy patients aged between 18 and 50 years. To assess systemic inflammation, a composite pro-inflammatory score was derived from the expression of 12 pro-inflammatory proteins. Patients were stratified based on pro-inflammatory score and NEFL and correlation between them was analyzed. Seizure frequency and drug resistance were assessed across patient subgroups.

**Results:**

Pro-inflammatory score and NEFL showed weak positive correlation (*r* = 0.1); not all patients with high levels of inflammation had high levels of NEFL. In the small proportion of patients (11%, *n* = 19) with high inflammation and elevated NEFL, seizures (Kruskal–Wallis test *H* = 9.68, *p* = 0.02) and drug-resistant epilepsy (χ^2^ = 13.47, *p* = 0.036, df = 6) were more common, whereas patients with low inflammation and normal NEFL (31.8%, *n* = 56) tended to have well-controlled epilepsy. Moreover, patients with high inflammation and abnormal NEFL showed protein changes suggestive of potential blood–brain barrier disruption and leukocyte migration.

**Conclusions:**

Inflammation and neurodegeneration are not necessarily linked in all epilepsy patients, but both are more likely to exist in the subset of cases with many seizures. Conversely, absence of both processes indicates well-controlled epilepsy. The combination of plasma NEFL with inflammatory markers could improve seizure prediction and provide novel insights for personalized epilepsy management.

**Supplementary Information:**

The online version contains supplementary material available at 10.1186/s12916-025-04425-z.

## Background

Epilepsy is a common brain disorder affecting more than 50 million people of all ages worldwide and characterized by the occurrence of seizures. Although advancements have been made in both drug and surgical therapies for epilepsy, we still do not fully understand what causes seizures and mechanisms that change a healthy brain to epileptic. This gap in knowledge has hindered the development of new treatments and drug discovery, contributing to the fact that approximately 30% of patients remain resistant to current therapies [1]. Epilepsy is a highly heterogeneous condition, with variations in age of onset, seizure types, underlying causes, and comorbidities. This complexity creates additional significant challenges in identifying effective, one-size-fits-all treatment approaches. This highlights the need for precision medicine strategies to better classify patients and develop targeted therapies that improve outcomes. Epileptogenesis, the development of epilepsy, often seems to involve immune and inflammatory mechanisms [2]. The underlying pathophysiology of epilepsy remains incompletely understood, but evidence suggests that inflammation can play a role in its onset and progression [3]. Studies in both patients and animal models have revealed altered cytokine expression and immune cell function, indicating a complex interaction between the immune system and seizure activity. Pro-inflammatory molecules cause neuroinflammation, which triggers neuronal hyperexcitation that disrupts neuronal function, ultimately contributing to epileptogenesis and progression [4]. Prolonged seizures can, in turn, amplify inflammatory responses, potentially as part of a compensatory mechanism for tissue repair [5], but also exacerbating seizure severity [6]. Elevated levels of inflammatory cytokines, whether originating in the brain or peripheral blood, can compromise blood–brain barrier (BBB). This compromised barrier permits infiltration of peripheral immune cells such as monocytes and lymphocytes into the brain. The infiltration of monocytes into brain tissues, such as the hippocampus, has been observed in both human and animal models of epilepsy [7]. This infiltration is associated with an increased expression of inflammatory markers and has been linked to neuronal damage, particularly in conditions like temporal lobe epilepsy (TLE) and hippocampal sclerosis [7, 8]. Various omics-based approaches—transcriptomics, proteomics, and epigenetics—using patient and animal model samples (brain, cerebrospinal fluid (CSF), and blood) consistently highlight the central role of inflammation in epileptogenesis. Single-cell RNA-seq of epileptic brain tissues identified distinct epilepsy-associated neuronal subtypes and pro-inflammatory microenvironment [9, 10]. Proteomic studies across multiple epilepsy models and human tissues have revealed shared enrichment of immune and inflammatory pathways [11]. Furthermore, network-based protein interaction analyses have identified modules containing inflammatory markers linked to seizure activity [12]. Epigenetic analyses also reveal significant DNA methylation changes in epilepsy, particularly in the hippocampus and anterior temporal neocortex, affecting genes associated with neuroinflammatory signaling [13].

Neurodegeneration is another key pathophysiological process in epilepsy. Neurofilament light chain (NEFL or NfL) is a peripheral biomarker for neurodegeneration [14]. This neuron-specific protein is a key component of the axonal cytoskeleton and involved in axonal growth and stability [15]. Under physiological conditions, NEFL levels in CSF and blood remain low. However, neuronal damage causes a significant increase in NEFL, making it a reliable indicator of axonal injury [16]. Elevated levels of NEFL have been connected to cognitive decline [17], structural brain alterations [18], dementia [19], and aging [20]. Prolonged seizures can damage neurons and disrupt the BBB, releasing neuronal proteins such as NEFL in the blood [21].

In the last years, blood biomarkers of inflammation and neurodegeneration have been increasingly reported in epilepsy. Seizures induce inflammation, which can be detectable through blood cytokines [12, 22]. Severe seizures cause higher serum NEFL [21]. Most patients with epilepsy do not have biomarkers suggesting ongoing neurodegeneration, but elevated levels are more common in those with frequent seizures or cognitive symptoms [23]. This raises interesting biological questions: what characterizes epilepsy with inflammation and no evidence of degeneration or vice versa? We used a large Olink dataset of neurological and immunological markers to characterize the association between NEFL and pro-inflammatory proteins with specific focus on features of epilepsy in patients with evidence of both, either, or none of these biological processes. Here we show that the extent of inflammation and neurodegeneration varies across epilepsy patients, resulting in distinct patient groups. Those with both high inflammation and elevated NEFL were more likely to experience frequent seizures and drug-resistant epilepsy, whereas individuals with low inflammation and normal NEFL were more likely to have well-controlled epilepsy. These results highlight the potential for stratifying patients based on inflammatory and neurodegenerative profiles, which could improve patient classification and guide the development of targeted therapies.

## Methods

### Study design and participant selection

Patients with epilepsy were selected from the “Prospective Regional Epilepsy Database and Biobank for Individualized Clinical Treatment (PREDICT)” study (registered at clinicaltrials.org, NCT04559919). Recruitment to PREDICT started in December 2020 at five neurology outpatient clinics within the Västra Götaland region of Sweden. The study has received approval from the Swedish Ethical Review Authority (approval number 2020–00853). All participants provided written informed consent prior to inclusion. A total of 176 participants aged 18–50 years, all diagnosed with epilepsy according to the latest International League Against Epilepsy (ILAE) criteria, were included in this study [24]. Details of clinical characteristics were collected into a pseudonymized clinical report form (CRF) (age, gender, seizure frequency, and types of epilepsy) and were reported previously [22]. Patients were categorized as “seizure-free” if no seizures for > 1 year, “recent seizures” if seizures occur ≤ 2 months, and “seizure for > 2 months to ≤ 1 year.” Patients were categorized as “drug-resistant epilepsy (DRE)” if they were on two or more anti-seizure medications (ASMs) and had seizures in the last year. Those who were on one ASM and had no seizures for > 1 year were categorized as well-controlled epilepsy (WCE). Remaining patients who did not meet the criteria for either DRE or WCE—including those not on ASMs—were categorized as undetermined. To classify patients by epilepsy type, we first relied on the clinician’s classification in the medical notes. If that was missing, then we used clinical data available at the time of data extraction including age of onset, seizure status, and imaging results. Based on these criteria, patients were categorized as focal, generalized, or epilepsy of unknown cause. Subclassification into subsyndromes like TLE or JME were relatively rare in medical records.

### Blood collection and storage

Blood samples for the PREDICT study were drawn into EDTA tubes at the time of recruitment. These samples were centrifuged at 2000 × g for 10 min at room temperature, after which the plasma was separated, aliquoted, and stored at − 80 °C in the regional Biobank Väst (registration number 890) for subsequent analyses [22].

### Olink proteomics

Plasma proteomics analysis was performed at SciLifeLab Uppsala (Uppsala University, Sweden) using four Olink Explore 3072/384 panels provided by Olink (Uppsala, Sweden). The panels included Neurology I, Neurology II, Inflammation I, and Inflammation II. Olink Explore detects and quantifies proteins in every sample. Olink employs a dual-recognition immunoassay based on the Proximity Extension Assay (PEA) technology. This method requires two oligonucleotide-linked antibodies, referred to as PEA probes, to bind near their target protein. Upon binding, the oligonucleotides hybridize to form a unique DNA template, which is subsequently detected through sequencing. Results are reported as Normalized Protein eXpression (NPX) values, presented as arbitrary units on a log_2_ scale for relative quantification. To accommodate the wide range of plasma protein concentrations, each Explore 384 panel is divided into four dilution blocks, with 0.2 µL of undiluted or diluted plasma added per well. The Olink NPX pipeline automatically corrects for dilution factors. Sample handling and assay miniaturization were performed using calibrated nanoliter liquid handlers to ensure reproducibility. Quality control included both internal and external controls. Internal controls (incubation, extension, and amplification) were spiked into every sample to monitor assay and sample performance. QC flags were generated automatically by the Olink NPX software in cases of technical issues such as sample volume discrepancies.

### Data preprocessing

Preprocessing of Olink data is reported previously [22]. Briefly, the four Olink panels were combined. To identify and address the duplicate protein (assays) entries across the four Olink panels, a pairwise comparison between the panels was conducted. Three proteins, namely, TNF, IL6, and CXCL8, were found to be duplicated between the Inflammation and Neurology panels, while LMOD1, SCRIB, and IDO1 were duplicated between the Inflammation_II and Neurology_II panels. For each set of duplicates, the version with the highest geometric mean expression was retained. This approach ensured that the selected proteins were those with expression levels relatively higher than the limit of detection (LOD), therefore maximizing data quality and reliability in the final merged dataset. Nineteen proteins that failed to meet Olink’s batch release quality control standards were excluded. The resulting dataset included 1447 proteins. Further analyses using these proteins were conducted in Python 3.12.4.

### Selection of pro-inflammatory proteins

A systematic literature search was conducted to select those pro-inflammatory proteins which have been shown to be upregulated in plasma or serum of epilepsy patients or after seizure in at least 2 publications. The following query was used to filter relevant literature from PubMed: “(seizure [Title/Abstract] OR epilepsy [Title/Abstract]) AND (gene symbol [Title/Abstract]) AND (serum [Title/Abstract] OR plasma [Title/Abstract]).” This search identified the following 12 pro-inflammatory proteins: CASP1, CCL11, CCL2, CCL3, CXCL8, IFNG, IL17A, IL18, IL1B, IL2, IL6, TNF.

### Calculation of Z-score and composite pro-inflammatory score (PI_Score)

To create a composite pro-inflammatory protein score, Z-scores for each of the above identified 12 proteins were calculated using the following formula: Z-score = (individual NPX − mean NPX)/Standard Deviation. Then, all the 12 Z-scores were added to create a composite pro-inflammatory score for each patient. Patients were stratified into four quadrants by plotting NEFL expression from Olink along with pro-inflammatory score. A cutoff of 0.5 NPX was chosen for NEFL based on quantification of NEFL in same patients via single molecule array (Simoa) N4PB kit (Quanterix) method [23]. NEFL 0.5 NPX corresponds to 10 pg/mL cutoff above which NEFL levels are abnormal for the age range 18–50 years (Additional file 1: Fig. S1A) [20]. To define groups with relatively low and high pro-inflammatory activity, samples below the 40th percentile of the PI_Score were classified as “low inflammation” and those above the 60th percentile as “high inflammation.” This percentile-based approach provides biological contrast by excluding the ambiguous central 20% patients (those close to the median) to reduce classification noise. Unclassified patients between 40 and 60th percentile of the PI_Score were excluded from further analysis, ensuring focused analysis on the remaining quadrants.

### Statistical analysis

Descriptive statistics were computed for each quadrant, including the number of patients, mean, standard deviation, minimum, and maximum values of the variable. For continuous variables, a non-parametric Kruskal–Wallis *H* test was performed in Python to compare the distribution of variables across different quadrants. Following the Kruskal–Wallis *H* test, we performed post hoc pairwise comparisons using Dunn’s test with a Bonferroni correction for multiple comparisons, to identify which specific quadrants significantly differed from one another.

For categorical variables, the chi-square test was performed in Python to examine the association between the variable and the quadrants. Specifically, the chi-square test of independence was applied to the contingency table to test the null hypothesis that there was no association between the categorical variable and the quadrant. If the chi-square test is significant, post hoc pairwise chi-square test with Benjamini–Hochberg correction was performed for that variable. The test statistics, degrees of freedom, and the associated *p* value were reported. Results were considered statistically significant if the *p* value was less than 0.05.

### Correspondence analysis (CA) and Euclidean distance calculation

To explore the association between categorical variables and quadrant-based classification, we conducted a CA followed by Euclidean distance calculations. The analysis was conducted using the prince package in Python with two principal components selected to capture the most meaningful variation in the data [25]. First, a contingency table was created to compute the frequency of each category of variable within the quadrants. Next, this table is decomposed into a set of coordinates that best represent the association between rows and columns. This is achieved through singular value decomposition (SVD) of the standardized residual matrix. The resulting principal coordinates map the categorical variables into a lower-dimensional space while preserving their relationships. Finally, these coordinates were used for Euclidean distance calculation between each quadrant and the corresponding categories for visualization.

### Volcano plot generation

A volcano plot was generated using custom Python script incorporating the pandas, numpy, matplotlib, and seaborn libraries to visualize differentially expressed proteins between the “High NEFL–High PI_Score” group and the “Normal NEFL–Low PI_Score” group. Log_2_ fold changes and *p* values were used to assess differential expression. Multiple testing correction was performed using the Benjamini-Hochberg (BH) method to obtain false discovery rate (FDR)-adjusted *p* values. To highlight significance, proteins were categorized as significant if FDR-adjusted *p* value < 0.05, raw *p* value < 0.05, and |log_2_ fold change|≥ 1. The y-axis was broken, and a portion of the graph was zoomed in to improve visualization of significant upregulated proteins. The top 20 most differentially expressed proteins were labeled on the plot.

### Functional association network analysis

Network analysis and enrichment of biological processes were performed using GeneMANIA (https://genemania.org/) [26]. To avoid bias, proteins used to calculate the PI_Score and NEFL were excluded and 19 significantly upregulated proteins from “High-High” quadrant were provided as input. The settings included a maximum of 10 resultant genes and 10 resultant attributes, with the network weighted using the automatically selected method. The top five functions based on FDR are shown in the figure.

## Results

### Cohort description

Our study population included 176 epilepsy patients with a median age of 31 years (range: 18–50). There were no cases of acquired lesions caused by stroke, trauma, or similar events among the patients. Of the total cohort, 84 were diagnosed with focal epilepsy, 49 with generalized epilepsy, and 43 with epilepsy of unknown etiology. Based on seizure status, 88 patients were seizure-free, 71 had recent seizures, and 17 patients were in “seizure for > 2 months to ≤ 1 year” category. Seizure frequency data was available for 170 patients ranging from 0 to 98 seizures (Table 1).

**Table 1:** Description of the study cohort

	**Total (*n* = 176)**
**Median age (range)**	31 (18–50) years
**Gender**	
Male	73 (41.5%)
Female	103 (58.5%)
**Epilepsy type**	
Focal	84 (47.7%)
Generalized	49 (27.8%)
Unknown	43 (24.4%)
**Seizure status**	
Seizure-free (> 1 year)	88 (50.0%)
Recent seizures (≤ 2 months)	71 (40.3%)
Seizures > 2 months– ≤ 1 year	17 (9.7%)
**Median seizure frequency (range)**	0 (0–98), data available for *n* = 170
**Current no. of anti-seizure medications (ASMs)**	
0	16 (9.0%)
1	98 (55.7%)
2	41 (23.3%)
3 +	21 (11.9%)

### Literature-based protein selection for pro-inflammatory score

**Fig. 1:**
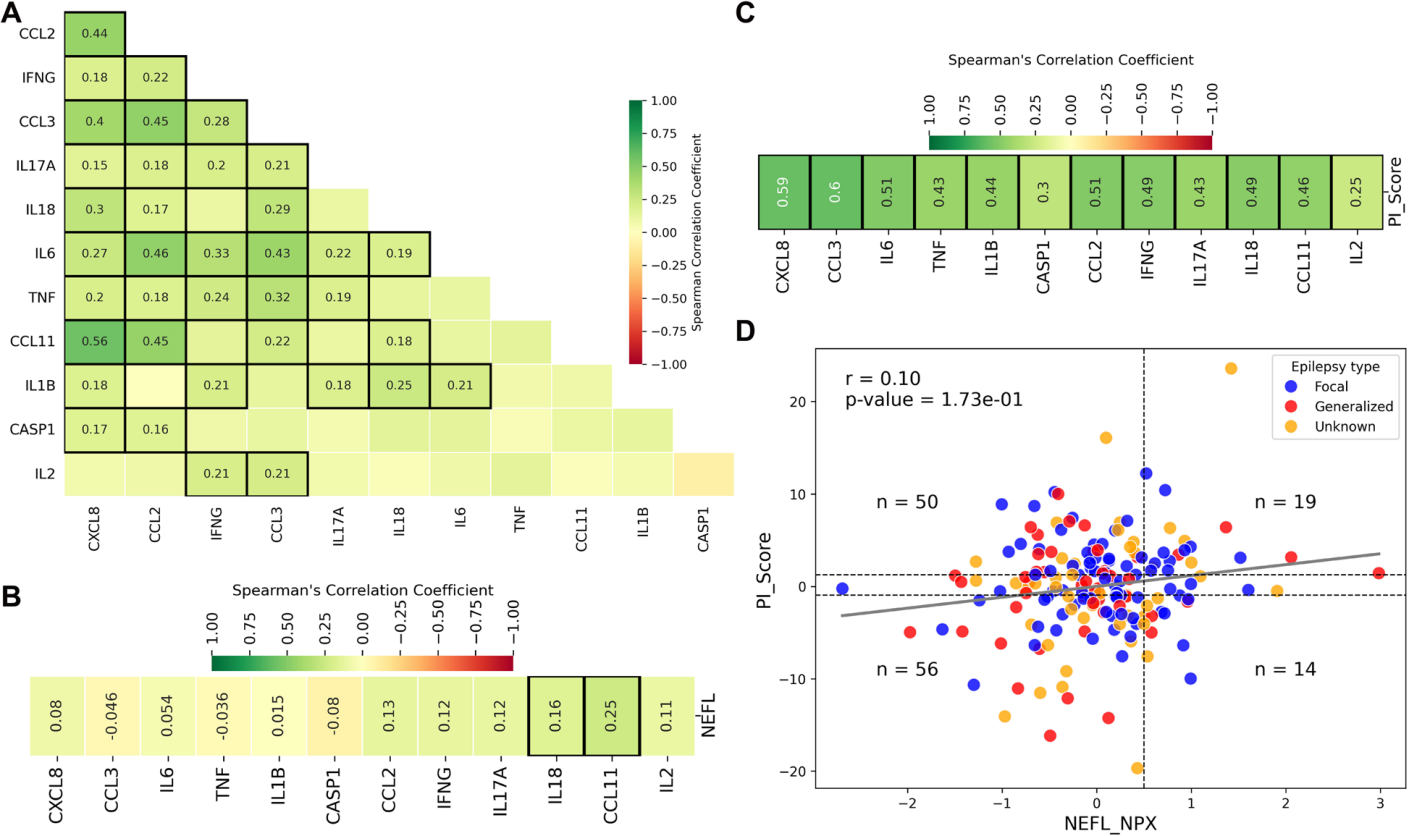
Correlation between plasma NEFL and inflammation in the epilepsy cohort. **A** Correlation plot of inflammation-associated proteins. Plasma protein levels, obtained from Olink proteomics of epilepsy patients, were used to calculate Spearman’s correlation coefficients between the proteins. Proteins with correlation coefficients |*r*|> 0.1 and *p* values < 0.05 are highlighted with black borders. **B** Correlation plot of inflammation-associated proteins with NEFL is shown as described in **A**. **C** Correlation plot of inflammation-associated proteins with PI_Score is shown as described in **A**. **D** Scatter plot of NEFL and PI_Score. The vertical dotted line at x = 0.5 NPX corresponds to 10 pg/mL, representing threshold above which plasma NEFL levels are reported as abnormal. Horizontal dotted lines plotted with 40th and 60th percentile cutoffs to separate patients with high inflammation from those with low inflammation. Patients between the dotted lines are unclassified and excluded from further analysis. The gray regression line represents the best-fit line modeling the relationship between the PI_Score and NEFL. *r* = Spearman’s correlation coefficients, *n* = number of patients in each quadrant

A systematic literature search was conducted in PubMed to find pro-inflammatory proteins upregulated in serum or plasma after seizure or in epilepsy. From 94 inflammation-associated proteins from Olink data including cytokines, chemokines, and their receptors, of which 74 were pro-inflammatory, 12 proteins were identified as being upregulated in serum/plasma of at least two cohort studies of seizure/epilepsy patients. These proteins include CASP1, CCL11, CCL2, CCL3, CXCL8, IFNG, IL17A, IL18, IL1B, IL2, IL6, and TNF. Spearman correlation analysis among these proteins revealed significant positive correlations in our patient cohort, except for CASP1 and IL2, which exhibited the lowest correlation with other proteins (Fig. 1A). Interestingly, NEFL was not correlated with many of these proteins. NEFL only showed a weak positive correlation with IL18 and CCL11 in our cohort (Fig. 1B). Combined Z-score from these 12 proteins gave a pro-inflammatory score (PI_Score) for each patient (Additional file 2: Table S1). As expected, the PI_Score was significantly and positively correlated with all the 12 proteins used in its calculation (Fig. 1C). Importantly, it was also significantly and positively correlated with 43 out of the remaining 62 pro-inflammatory proteins that were not included in the PI_Score calculation, supporting its validity as a marker of systemic inflammation (Additional file 1: Fig. S1B). In contrast, NEFL exhibited significant positive correlations with only five pro-inflammatory proteins and a negative correlation with one out of 62, underscoring its weak association with inflammatory pathways (Additional file 1: Fig. S1C).

### Patient categorization

Our newly constructed PI_Score was plotted against NEFL to allow categorization of patients in four quadrants, namely, “Normal NEFL-Low PI_Score” (*n* = 56), “High NEFL-High PI_Score” (*n* = 19), “Normal NEFL-High PI_Score” (*n* = 50), and “High NEFL-Low PI_Score” (*n* = 14) (Fig. 1D, Additional file 2: Table S1). The age distribution across the four quadrants differed significantly, with patients in the “Normal NEFL-Low PI_Score” group being younger (mean age 30.9 years) compared to those in the “High-High” quadrant (mean age 39.6 years) (Kruskal–Wallis test, *p* = 0.02, Table 2). However, age at onset and epilepsy duration did not significantly vary among the quadrants (Table 2). NEFL showed weak positive correlation with PI_Score (*r* = 0.1) in our cohort. NEFL levels and the PI_Score was comparable across patients with focal, generalized, and unknown epilepsy type (χ^2^ = 2.14, *p* = 0.90, df = 6) (Fig. 1D, Table 3).

**Table 2:** Descriptive statistical analysis for age, seizure frequency, epilepsy duration, and age at onset in each quadrant, Kruskal–Wallis test to check association between continuous variables (age, seizure frequency, epilepsy duration, age at onset) and quadrants, and Dunn’s test with post hoc Bonferroni correction for pairwise comparisons between quadrants. *N* = number of patients in each group, std dev = standard deviation, *H* = Kruskal–Wallis *H* statistic, *p* = *p* value

	**Quadrants**	**Descriptive statistics**					**Kruskal–Wallis test**		**Dunn’s test post hoc results (with Bonferroni correction)**			
		***N***	**Mean**	**std dev**	**Min**	**Max**	***H***	***p***	**Normal (NEFL)-Low (PI_Score)**	**High-High**	**Normal (NEFL)-High (PI_Score)**	**High (NEFL)-Low (PI_Score)**
**Age**	Normal (NEFL)-Low (PI_Score)	56	30.91	8.24	18	48	10.08	**0.02**	–	0.01	0.74	1
	High-High	19	39.63	11.72	20	50			0.01	–	0.30	0.40
	Normal (NEFL)-High (PI_Score)	50	33.30	8.75	18	50			0.74	0.30	–	1
	High (NEFL)-Low (PI_Score)	14	33.00	10.05	21	48			1.00	0.40	1	–
**Seizure frequency**	Normal (NEFL)-Low (PI_Score)	53	3.32	10.08	0	60	9.68	**0.02**	–	0.01	1.00	1.00
	High-High	19	9.53	14.93	0	45			0.01	–	0.10	0.50
	Normal (NEFL)-High (PI_Score)	49	2.61	9.13	0	60			1.00	0.10	–	1.00
	High (NEFL)-Low (PI_Score)	14	10.50	26.94	0	98			1.00	0.50	1.00	–
**Epilepsy duration**	Normal (NEFL)-Low (PI_Score)	53	12.32	10.14	0	47	3.48	0.32	–	–	–	–
	High-High	19	17.32	11.77	0	41			–	–	–	–
	Normal (NEFL)-High (PI_Score)	42	12.55	12.18	0	42			–	–	–	–
	High (NEFL)-Low (PI_Score)	14	14.43	15.70	0	47			–	–	–	–
**Age at onset**	Normal (NEFL)-Low (PI_Score)	53	18.94	11.59	0	45	1.12	0.77	–	–	–	–
	High-High	19	22.32	12.73	4	48			–	–	–	–
	Normal (NEFL)-High (PI_Score)	42	19.55	11.16	1	46			–	–	–	–
	High (NEFL)-Low (PI_Score)	14	18.57	11.93	0	38			–	–	–	–

Numbers in bold indicate statistically significant p-values (*p*<0.05)

**Table 3:** Statistical analysis. Chi-square (χ^2^) test to analyze relationships between categorial variables (seizure status, epilepsy type, gender, epilepsy status, number of ASMs) with quadrants. *p* = *p* value, df = degrees of freedom. When χ^2^ test is significant, post hoc pairwise χ^2^ test performed with Benjamini-Hochberg (BH) correction. *DRE* drug-resistant epilepsy, *WCE* well-controlled epilepsy, *ASMs* anti-seizure medications

	**Quadrant**	**Recent seizure**		**Seizure > 2 months to ≤ 1 year**		**Seizure-free for > 1 year**		**χ**^**2**^	***p***	**df**				
**Seizure status**	High (NEFL)-Low (PI_Score)	6		2		6		12.34	0.055	6				
	High-High	13		3		3								
	Normal (NEFL)-High (PI_Score)	20		7		23								
	Normal (NEFL)-Low (PI_Score)	18		4		34								
	**Quadrant**	**Focal**		**Generalized**		**Unknown**		**χ**^**2**^	***p***	**df**				
**Epilepsy type**	High (NEFL)-Low (PI_Score)	6		3		5		2.14	0.907	6				
	High-High	11		4		4								
	Normal (NEFL)-High (PI_Score)	26		14		10								
	Normal (NEFL)-Low (PI_Score)	27		16		13								
	**Quadrant**	**Male**		**Female**				**χ**^**2**^	***p***	**df**				
**Gender**	High (NEFL)-Low (PI_Score)	10		4				5.85	0.119	3				
	High-High	8		11										
	Normal (NEFL)-High (PI_Score)	22		28										
	Normal (NEFL)-Low (PI_Score)	20		36										
	**Quadrant**	**DRE**		**Undetermined**		**WCE**		**χ**^**2**^	***p***	**df**	**Post hoc pairwise chi-square test comparison groups**		**Original *p* value**	**Corrected *p* value**
**Epilepsy status**	High (NEFL)-Low (PI_Score)	4		4		6		13.47	**0.036**	6	High-High vs Normal (NEFL)-High (PI_Score)		**0.008**	**0.03**
	High-High	11		5		3					High-High vs Normal (NEFL)-Low (PI_Score)		**0.005**	**0.03**
	Normal (NEFL)-High (PI_Score)	10		21		19					High-High vs High (NEFL)-Low (PI_Score)		0.16	0.31
	Normal (NEFL)-Low (PI_Score)	11		20		25					Normal (NEFL)-High (PI_Score) vs Normal (NEFL)-Low (PI_Score)		0.76	0.76
											Normal (NEFL)-High (PI_Score) vs High (NEFL)-Low (PI_Score)		0.63	0.76
											Normal (NEFL)-Low (PI_Score) vs High (NEFL)-Low (PI_Score)		0.74	0.76
	**Quadrant**	**0**	**1**	**2**	**3**	**4**	**5**	**χ**^**2**^	***p***	**df**		High-High vs Normal (NEFL)-High (PI_Score)	0.18	0.33
**Number of ASMs**	High (NEFL)-Low (PI_Score)	1	9	2	1	0	1	27.1	**0.028**	15		High-High vs Normal (NEFL)-Low (PI_Score)	**0.02**	0.14
	High-High	2	6	6	3	2	0					High-High vs High (NEFL)-Low (PI_Score)	0.28	0.33
	Normal (NEFL)-High (PI_Score)	7	29	9	4	1	0					Normal (NEFL)-High (PI_Score) vs Normal (NEFL)-Low (PI_Score)	0.17	0.33
	Normal (NEFL)-Low (PI_Score)	2	34	17	3	0	0					Normal (NEFL)-High (PI_Score) vs High (NEFL)-Low (PI_Score)	0.49	0.49
												Normal (NEFL)-Low (PI_Score) vs High (NEFL)-Low (PI_Score)	0.24	0.33

Numbers in bold indicate statistically significant p-values (*p*<0.05)

### High inflammation and increased plasma NEFL associate with seizures

**Fig. 2:**
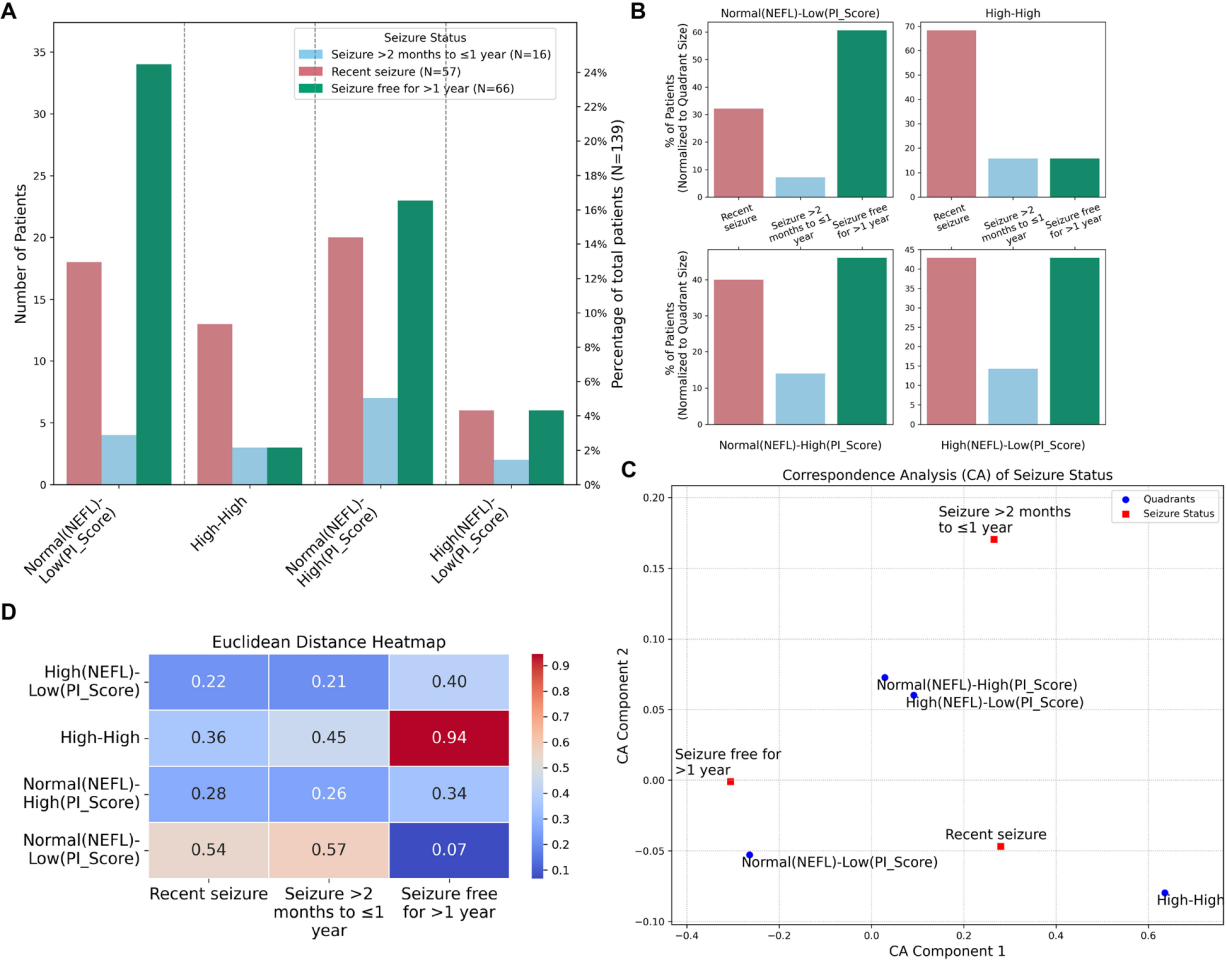
High inflammation and increased NEFL associate with more seizures. **A** Patients were grouped into four quadrants based on NEFL and PI_Score. The bar plot shows patient distribution in each quadrant (excluding the “unclassified”) according to their seizure status. **B** Bar plot showing the percentage of patients with each seizure status within a quadrant, relative to the total number of patients in that quadrant. **C** Correspondence analysis (CA) scatter plot to show the relationship between quadrants and categorical variable (seizure status) based on the first two CA components. The closer a quadrant point is to a category point, the stronger the association between them. **D** Euclidean distance heatmap quantifying the proximity between quadrants and seizure status category

Seizure status distribution across quadrants showed that among patients in the “Normal NEFL-Low Inflammation” quadrant, 59% were seizure-free (33 out of 56 patients in that quadrant). This quadrant contained 33 out of the 66 total quadrant classified seizure-free patients, the highest among all quadrants (Fig. 2A, B). The “High-High” quadrant had the highest proportion of patients with seizures (68%, 13 out of 19 patients in that quadrant) (Fig. 2B). In the “Normal NEFL and High Inflammation” quadrant, the number of recent seizure patients was nearly equal to the number of seizure-free patients (Fig. 2A). Similarly, the “High NEFL and Low Inflammation” quadrant also showed a comparable distribution of recent seizure and seizure-free patients (Fig. 2A). The distribution of seizure status across quadrants was similar between focal and generalized epilepsy, except in the “Normal NEFL–High Inflammation” quadrant. In this quadrant, focal epilepsy patients were more likely to have recent seizures, whereas generalized epilepsy patients were more often seizure-free (Additional file 1: Fig. S2A, B).

Correspondence analysis (CA) revealed an association between seizure status and the four quadrants. The seizure-free category was closest to the “Normal NEFL-Low Inflammation” quadrant, with a Euclidean distance of 0.07, indicating a strong association (Fig. 2C, D). In contrast, the seizure-free category was furthest from the “High-High” quadrant (Euclidean distance = 0.94) (Fig. 2C, D). Similarly, the recent seizure category showed the greatest distance from the “Normal NEFL-Low Inflammation” quadrant (Euclidean distance = 0.54) (Fig. 2C, D).

**Fig. 3:**
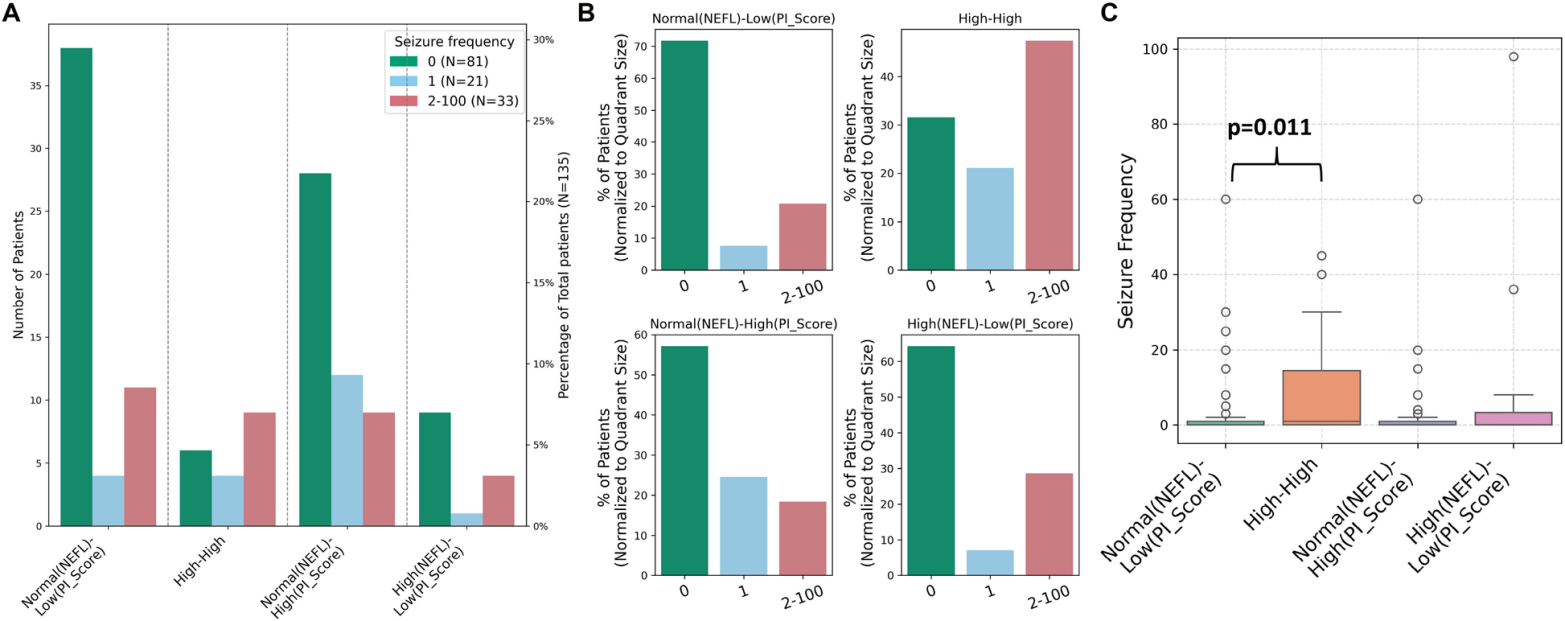
Low inflammation and normal NEFL are associated with seizure freedom. **A** Patients were divided into four quadrants based on NEFL and PI_Score. The bar plot shows patient distribution in each quadrant (excluding the “unclassified”) according to the seizure frequency. **B** Bar plot showing the percentage of patients with each seizure frequency category within a quadrant, relative to the total number of patients in that quadrant. **C** Seizure frequency analysis in four quadrants. Kruskal–Wallis test was used to assess differences in seizure frequency during the 2 months prior to inclusion among the quadrants (*p* = 0.02); *p* value on the plot denotes the result of Dunn’s post hoc test with Bonferroni correction for pairwise comparisons between “Normal (NEFL)-Low (PI_Score)” and “High-High” quadrants

Next, we analyzed patient distribution across four quadrants according to the patient-reported seizure frequency. Interestingly, 37 of 81 patients with 0 seizures were in the “Normal NEFL-Low Inflammation” quadrant (Fig. 3A), which had the highest proportion (70%) of seizure-free patients (Fig. 3B). The “High-High” quadrant showed a comparable distribution of seizure frequency groups (0, 1, 2–100 seizures) (Fig. 3B). In the “Normal NEFL and High Inflammation” and “High NEFL and Low Inflammation” quadrants, seizure-free patients were the largest group (Fig. 3A, B). The distribution of seizure frequency differs significantly across four quadrants (Kruskal–Wallis test, p = 0.02). Post hoc Dunn’s test with Bonferroni correction revealed a significant difference between the “Normal NEFL-Low Inflammation” and “High-High” quadrants (p = 0.011) (Table 2) (Fig. 3C). Patients in “High-High” quadrants had higher seizure frequency than patients in “Normal (NEFL)-Low (PI_Score)” quadrant.

### High inflammation and increased NEFL associate with drug-resistant epilepsy

**Fig. 4:**
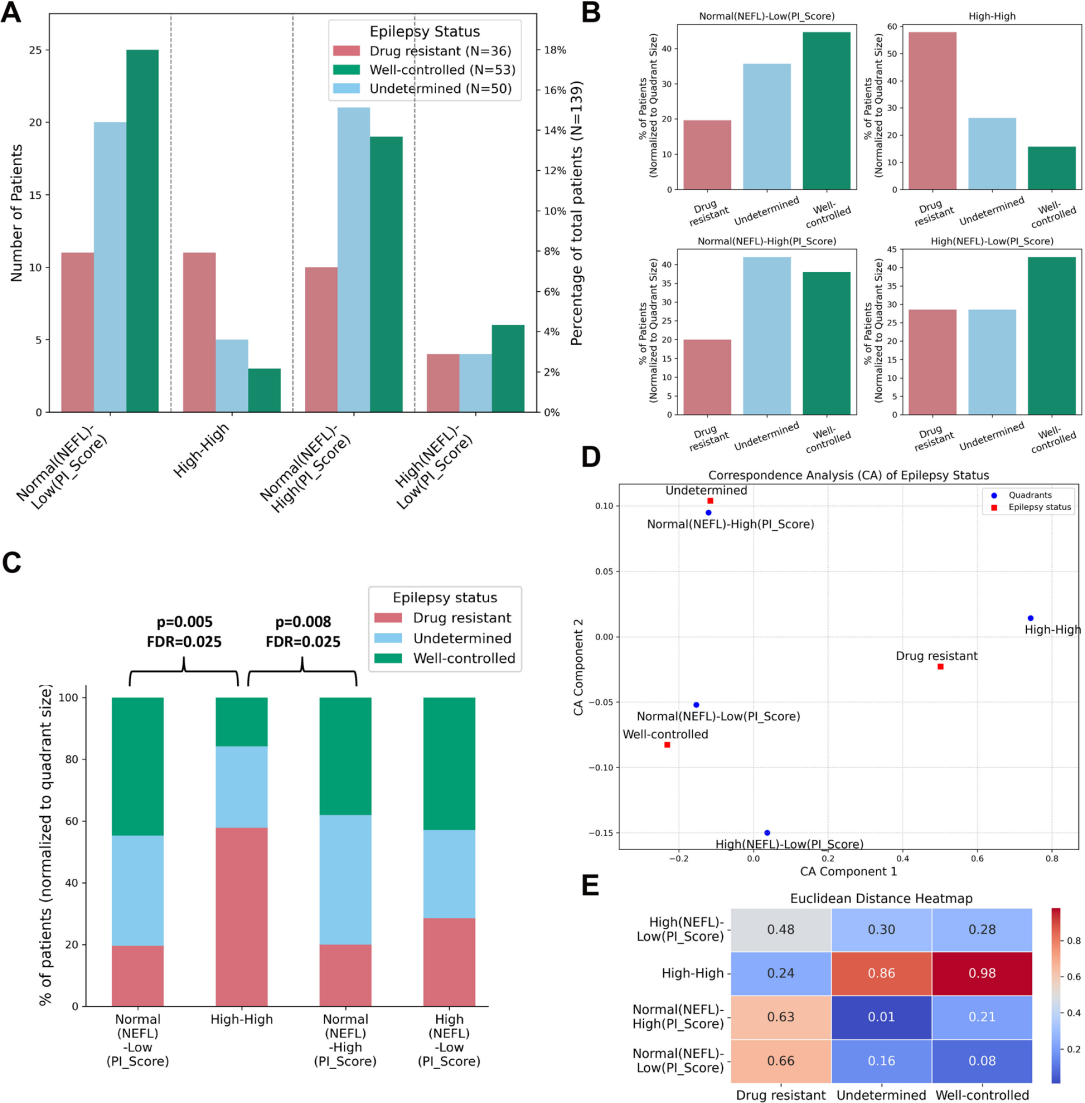
High inflammation and increased NEFL are linked to drug-resistant epilepsy. **A** Patients were grouped into four quadrants based on NEFL and PI_Score. The bar plot displays the number of patients in each quadrant (excluding the “unclassified”) by epilepsy status. **B** Bar plot showing the percentage of patients with each epilepsy status within a quadrant, relative to the total number of patients in that quadrant. **C** Stacked bar plot showing the distribution of epilepsy status groups across four quadrants, patient number normalized to the quadrant size. The *p* value is from post hoc pairwise χ^2^ test, and FDR denotes Benjamini–Hochberg correction applied to the *p* value. **D** Correspondence analysis (CA) scatter plot to show the relationship between quadrants and epilepsy status based on the first two CA components. **E** Euclidean distance heatmap quantifying the proximity between quadrants and epilepsy groups

Patients were categorized into drug-resistant epilepsy, well-controlled epilepsy, or unknown status. The distribution of these groups across the four quadrants differed significantly (χ^2^ = 13.47, *p* = 0.036, df = 6). Among the 53 well-controlled patients, 25 were in the “normal NEFL and low inflammation” quadrant (Fig. 4A), representing the highest proportion of well-controlled patients (45%) within that quadrant (Fig. 4B). In contrast, the “High-High” quadrant had the highest proportion of drug-resistant epilepsy patients (58%). Post hoc pairwise χ^2^ test with FDR correction showed a significant difference between the “Normal NEFL, Low Inflammation” and “High-High” quadrants (*p* = 0.005, FDR = 0.025) (Fig. 4C, Table 3).

In patients with focal epilepsy, the distribution of epilepsy status across the quadrants closely mirrored the overall patient cohort (Additional file 1: Fig. S3A). However, for generalized epilepsy, the distribution differed from the combined patient cohort. In the “Normal NEFL, Low Inflammation” quadrant, generalized epilepsy patients showed a more balanced proportion of drug-resistant and well-controlled cases, along with a higher representation of patients with undetermined epilepsy status (Additional file 1: Fig. S3B). Notably, the “High-High” quadrant of generalized epilepsy exclusively contained drug-resistant epilepsy patients. The highest number of well-controlled epilepsy patients in the generalized epilepsy group was found in the “Normal NEFL and High Inflammation” quadrant. The correspondence analysis showed a strong association between the drug-resistant epilepsy and the “High-High” quadrant, with a Euclidean distance of 0.24, indicating that drug-resistant patients were predominantly represented in this group (Fig. 4D, E). Conversely, the “well-controlled” category was furthest from the “High-High” quadrant (Euclidean distance = 0.98), highlighting a clear distinction between these epilepsy groups.

### High inflammation and neurodegeneration are associated with upregulation of markers of blood–brain barrier dysfunction and leukocyte migration

**Fig. 5:**
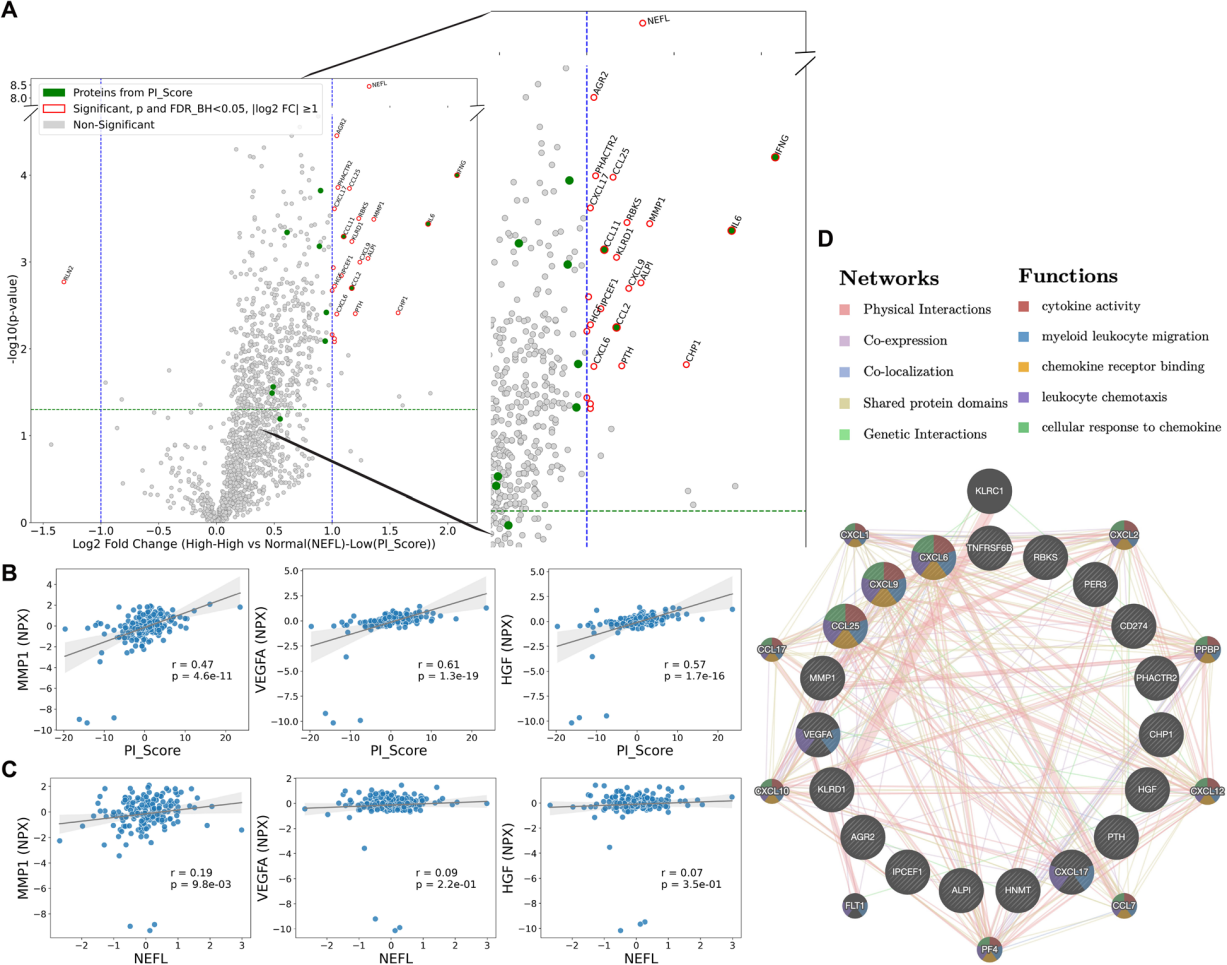
Differential expression analysis identified upregulation of blood–brain barrier-related proteins in patients with high inflammation and neurodegeneration. **A** Volcano plot showing differential protein expression analysis between the “High-High” and “Normal NEFL and Low Inflammation” groups. Proteins with an absolute log_2_ fold change ≥ 1, *p* < 0.05, and FDR_BH < 0.05 were depicted with red circles. Green points indicate the 12 proteins that were selected for pro-inflammatory score calculation. Top 20 significant proteins were annotated. The graph was zoomed in to improve visualization of significant upregulated proteins. Spearman correlations between BBB-associated proteins and **B** PI_Score and **C** NEFL. Gray line is for linear regression, and shaded areas represent 95% confidence intervals. *r*: Spearman correlation coefficient, *p*: *p* value. **D** Functional association network analysis of significantly upregulated proteins (*N* = 19) in “High-High” group using GeneMANIA (https://genemania.org/). Input (query) proteins are indicated by striped inner nodes. The top five enriched functional terms (based on FDR) are shown

Differential protein expression analysis between “High-High” and “Normal NEFL and Low Inflammation” quadrants revealed several significant protein level changes. As expected, the volcano plot showed higher expression of inflammatory proteins and NEFL in the “High-High” quadrant (Fig. 5A). Interestingly, proteins associated with BBB dysfunction were also significantly upregulated (log_2_ fold change ≥ 1 and *p* < 0.05) in this quadrant (Fig. 5A). These proteins include MMP1, VEGFA, and HGF. When using a relaxed cutoff (log₂ fold change > 0.5), additional BBB-related proteins—CXCL10, CXCL11, OCLN, and FGF2—were also significantly upregulated in the “High–High” group. Among these, matrix metalloproteinases 1 (MMP1) was highly upregulated top candidate with its distinct role in BBB breakdown. Furthermore, MMP1, VEGFA, and HGF showed significant positive correlations with PI_Score in our patient cohort (Fig. 5B). However, only MMP1 demonstrated a significant, but weak, positive correlation with NEFL (Fig. 5C). These findings suggest that inflammatory processes may actively contribute to the disruption of the BBB, which in turn may promote neurodegeneration and lead to elevated NEFL levels observed in patient plasma.

Next, functional network analysis was performed using 19 significantly upregulated proteins (CHP1, MMP1, ALPI, CXCL9, RBKS, PTH, KLRD1, CCL25, IPCEF1, PHACTR2, CXCL6, AGR2, TNFRSF6B, CXCL17, HGF, PER3, HNMT, CD274, VEGFA) in “High-High” quadrant, excluding those used to calculate the PI_Score and NEFL. This resulted in a dense network primarily connected through physical interactions, indicating strong functional associations among these proteins (Fig. 5D). The analysis revealed several enriched biological processes, including leukocyte migration, cytokine activity, and chemotaxis (Fig. 5D)—highlighting key immune and inflammatory pathways associated with patients in the “High-High” quadrant.

## Discussion

This study is part of a broader proteomics investigation aimed at exploring proteomic signatures across different clinical dimensions of epilepsy, including disease duration, inflammatory profiles, and epilepsy subtypes. The present analysis specifically focuses on the association between inflammation and neurodegeneration. Epilepsy is a complex neurological disorder in which both neuronal injury and inflammation are believed to contribute to disease progression. By leveraging plasma proteomics, we examined the relationship between inflammatory markers and NEFL, a marker of neurodegeneration, in patients with epilepsy. A pro-inflammatory score was derived from 12 pro-inflammatory proteins that were selected from the literature and known to be upregulated in epilepsy cohorts. Several of these proteins have been independently reported to be elevated across diverse epilepsy subtypes, reflecting the heterogeneity in immune responses. For instance, patients with mesial temporal lobe epilepsy (MTLE) have shown increased serum levels of CCL2, CCL3, and IL-8 compared to healthy controls [27]. Similarly, elevated levels of CASP1 and IL-1β have been associated with both febrile seizures and MTLE [28]. In severe forms like new-onset refractory status epilepticus (NORSE), pro-inflammatory cytokines such as IL-6, TNF-α, CXCL8/IL-8, and CCL2 are significantly upregulated compared to patients without status epilepticus [29]. Furthermore, seronegative autoimmune-associated epilepsy (snAAE) presents a distinct inflammatory signature, with elevated levels of 14 cytokines including CCL3, IL-6, and TNF [30]. Even within pediatric febrile seizures, IL-6 and IFN-γ levels are significantly higher than in afebrile seizure groups [31]. These findings underscore the presence of a shared inflammatory axis across epilepsy subtypes, despite clinical heterogeneity. The significant correlation of the PI_Score with both its constituent proteins and many additional pro-inflammatory markers suggests that it may reflect aspects of systemic inflammation across epilepsy phenotypes. Our analysis did not reveal a strong correlation between plasma NEFL levels and inflammation, suggesting that neurodegeneration and systemic inflammation may not always be linked in epilepsy, at least not in peripheral blood. When we categorized patients into groups based on both NEFL and pro-inflammatory score, distinct clinical patterns emerged. The observed distribution of seizure status across groups suggests a link between neurodegeneration, inflammation, and seizure control in a subgroup of patients. Individuals with elevated NEFL and high inflammation were more likely to experience frequent seizures and drug-resistant epilepsy, whereas those with normal NEFL and low inflammation were more likely to be seizure-free. Interestingly, previously it has been shown that patients with abnormally high levels of NEFL had more recent seizure activity. Specifically, NEFL levels were significantly higher in younger patients who had experienced seizures recently, compared to seizure-free [23]. These findings highlight the importance of considering both neuronal injury and inflammation in epilepsy, and that distinct pathophysiological mechanisms may be at work in different subsets of patients. Notably, our subgroup analysis stratifying patients by epilepsy types revealed important differences: a higher proportion of WCE cases was observed for generalized patients in the “Normal NEFL–High Inflammation” quadrant, where DRE cases were relatively few. In contrast, among patients with focal epilepsy, the “Normal NEFL–High Inflammation” quadrant contained more DRE than WCE cases. While the sample size within each subgroup limits the power for definitive statistical comparisons, this pattern suggests potential differences in how inflammation relates to seizure control across epilepsy types. It also suggests that the relationship between inflammation and seizure control may differ by epilepsy types—potentially indicating that systemic inflammation plays a different modulatory role in generalized versus focal epilepsy. However, due to the limited number of patients, particularly within the generalized epilepsy group, these observations should be interpreted cautiously. Future studies with larger, subtype-specific cohorts will be essential to determine whether inflammation truly has divergent implications for seizure control across epilepsy types.

Our finding further suggests that BBB dysfunction, leukocyte chemotaxis, and migration are prominent features in patients with high inflammation and neurodegeneration. Interestingly, BBB-associated protein were upregulated in this group. MMPs (matrix metalloproteinases) are known to be activated in the brain by epileptic seizures [32, 33]. Among them, MMP1 has an established role in BBB breakdown and emerged as a top candidate in our analysis. VEGFA, another upregulated protein, is well known for its pro-inflammatory effects, contributing to BBB dysfunction by increasing vascular permeability, attracting immune cells, and stimulating the release of other inflammatory mediators [34, 35]. Additionally, hepatocyte growth factor (HGF), which supports neuronal survival and has been implicated in neurodegenerative diseases, was also elevated [36, 37]. High HGF concentrations in CSF have been linked to accelerated cognitive decline [37], suggesting that its upregulation may reflect a compensatory mechanism aimed at protecting neurons from seizure-induced damage or alternatively, it could indicate a maladaptive process contributing to disease progression. The combined upregulation of these key BBB-associated proteins strongly supports the presence of BBB dysfunction in patients with high inflammation and neurodegeneration, which may further contribute to elevated NEFL levels in their plasma. While inflammation and neurodegeneration typically occur in distinct biological compartments, BBB disruption can facilitate cross-compartment interactions. NEFL, which is usually undetectable in the bloodstream, may enter circulation due to neuronal damage and BBB breakdown. Conversely, inflammatory cells, which are primarily present in the blood, can infiltrate the brain following BBB disruption, potentially exacerbating disease severity. Indeed, our gene ontology analysis suggest enhanced activation of leukocyte migration, chemotaxis, and cytokine activity in “High-High” group.

Our findings also suggest that inflammation could play a role in epilepsy severity for some patients with seizures, perhaps hinting at future possibilities of targeting immunotherapy for selected patient groups with drug-resistant epilepsy. The dissociation between NEFL and inflammatory markers also argues against inflammation being purely reactive to neurodegeneration resulting from seizures. Future research should focus on longitudinal studies to track how inflammation and neuronal injury evolve over time, as well as on identifying potential therapeutic strategies to mitigate their effects.

## Limitation

A key limitation of this study is relatively few individuals in specific subgroups of focal epilepsies, like TLE. A larger cohort size and comparisons to healthy control groups would have been informative. Although we focused on relatively homogeneous group of patients with only adults without acquired lesions, the heterogeneity in terms of different epilepsy syndromes, disease durations, and treatment histories may introduce biological variability that affects interpretation. For example, grouping patients with 2–100 seizures into a single category may mask clinically relevant differences across this range. While this binning helped maintain statistical power and simplify visualization, it represents a limitation that could influence interpretation of seizure burden across quadrants. We also note that this heterogeneity could partially explain why the “High-High” quadrant, despite showing a higher proportion of patients with recent seizures (Fig. 2), did not demonstrate clear differences in seizure frequency subgroups in Fig. 3B. Functional validation in an independent cohort would further strengthen the findings and provide additional mechanistic insights.

## Conclusions

Our findings indicate that plasma markers of inflammation and neurodegeneration were not necessarily associated in epilepsy and their relative levels varied across patients. While we observed a weak positive correlation between pro-inflammation and neurodegeneration, these processes were not universally linked. Some patients exhibited high inflammation without signs of neurodegeneration, while others showed elevated NEFL despite low inflammatory activity. Notably, the coexistence of both factors was associated with more frequent seizures and drug-resistant epilepsy, whereas their absence was linked to better seizure control. These findings suggest that while inflammation and neurodegeneration may independently contribute to epilepsy, their co-occurrence is more pronounced in patients with severe disease. Given the clinical and biological diversity of epilepsy, inflammation and neurodegeneration could serve as critical factors for stratifying patients into more precise subgroups. Identifying distinct inflammatory and neurodegenerative profiles may improve seizure prediction, refine treatment strategies, and enhance personalized approaches to epilepsy management.

## Supplementary Information

Additional file 1. Figures S1–S3. Fig. S1 Correlation analysis between proteins. Fig. S2 Seizure status analysis of focal and generalized patients. Fig. S3 Epilepsy status analysis of focal and generalized patients.

Additional file 2. Table S1 Z-scores and classification of patients in the quadrants: the NEFL (NPX), PI_Score, Z-scores for all the 1447 proteins and quadrant information is provided for all the patients.

## Data Availability

The datasets generated and/or analysed during the current study are not publicly available as the raw data of the study contain sensitive personal data and cannot be shared by the authors (according to Swedish research regulations).

## Abbreviations

NEFL: Neurofilament light chain
MMP1: Matrix metalloproteinases 1
HGF: Hepatocyte growth factor
NPX: Normalized Protein eXpression
CA: Correspondence analysis
BBB: Blood–brain barrier
PI_Score: Pro-inflammatory score
CSF: Cerebrospinal fluid
ILAE: International League Against Epilepsy
ASMs: Anti-seizure medications
FDR: False discovery rate
