# Supplementary material for: Association between pro-inflammatory proteins and neurofilament in plasma from persons with epilepsy
Source: BMC Med. 2025 Oct 13;23:554. doi: 10.1186/s12916-025-04425-z (PMC12519617; doi:10.1186/s12916-025-04425-z)
Supplement: Supplementary file 1 — Additional file 1. Figures S1–S3. Fig. S1 Correlation analysis between proteins. Fig. S2 Seizure status analysis of focal and generalized patients. Fig. S3 Epilepsy status analysis of focal and generalized patients. [file 12916_2025_4425_MOESM1_ESM.docx]

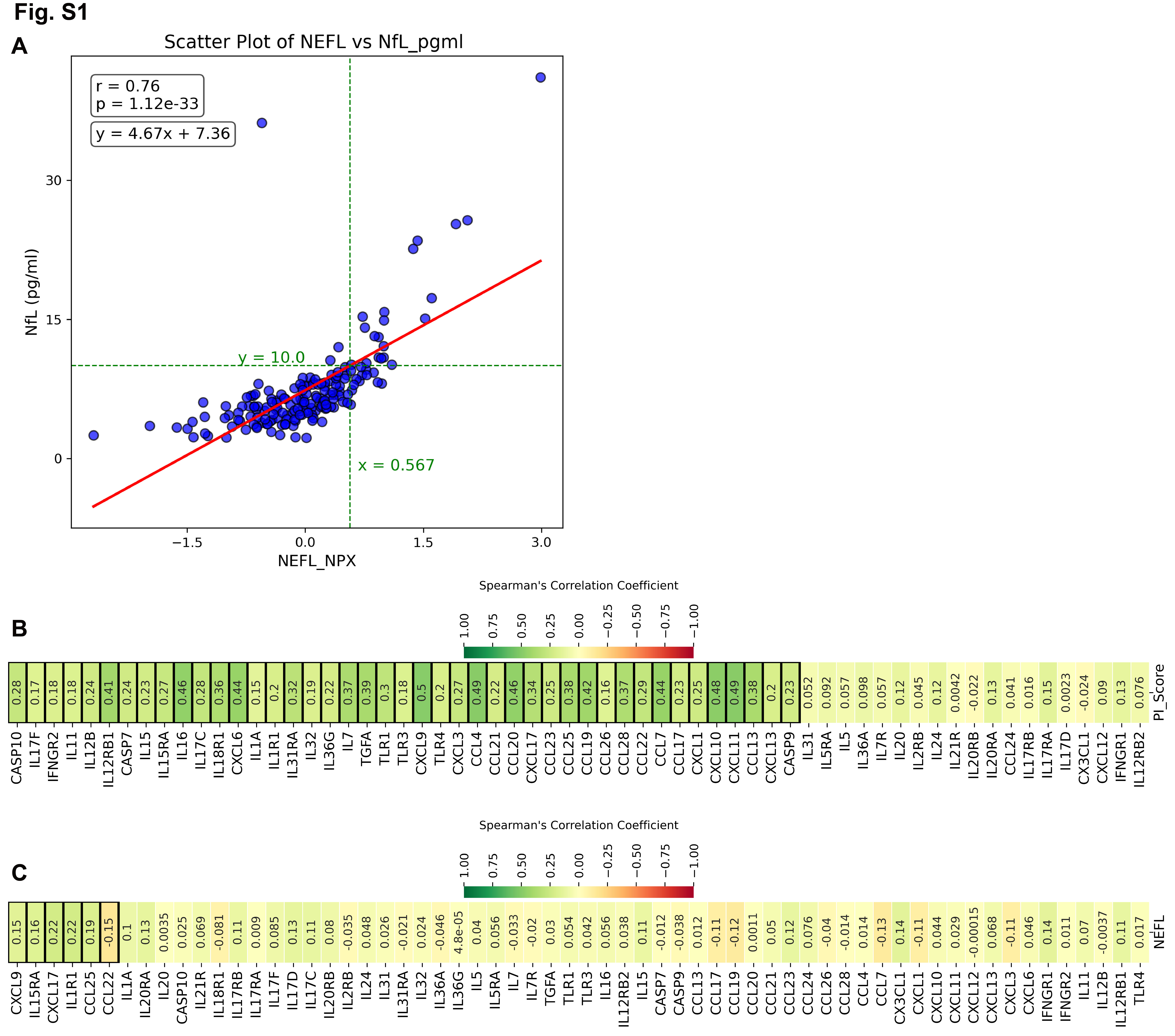


**Fig. S1: Correlation analysis between proteins.** **(A)** To derive actual concentration of NEFL in plasma, NPX values from Olink were correlated with NEFL expression measured by single molecule array (Simoa) N4PB kit (Quanterix) method in the same patients. A NEFL NPX value of 0.5 corresponds to a 10 pg/mL cutoff, above which NEFL levels are considered abnormal for individuals aged 18–50 years. r= spearman's correlation coefficient, p= p value. **(B-C)** Spearman correlations between pro-inflammatory proteins and (B) PI_Score and (C) NEFL. Out of the 94 inflammation associated proteins, 74 were pro-inflammatory. Of these, 12 proteins used to compute the PI_Score were excluded, and the remaining 62 proteins were used for the correlation analyses in both panels. Proteins with correlation coefficients |r| > 0.1 and p-values <0.05 are highlighted with black borders


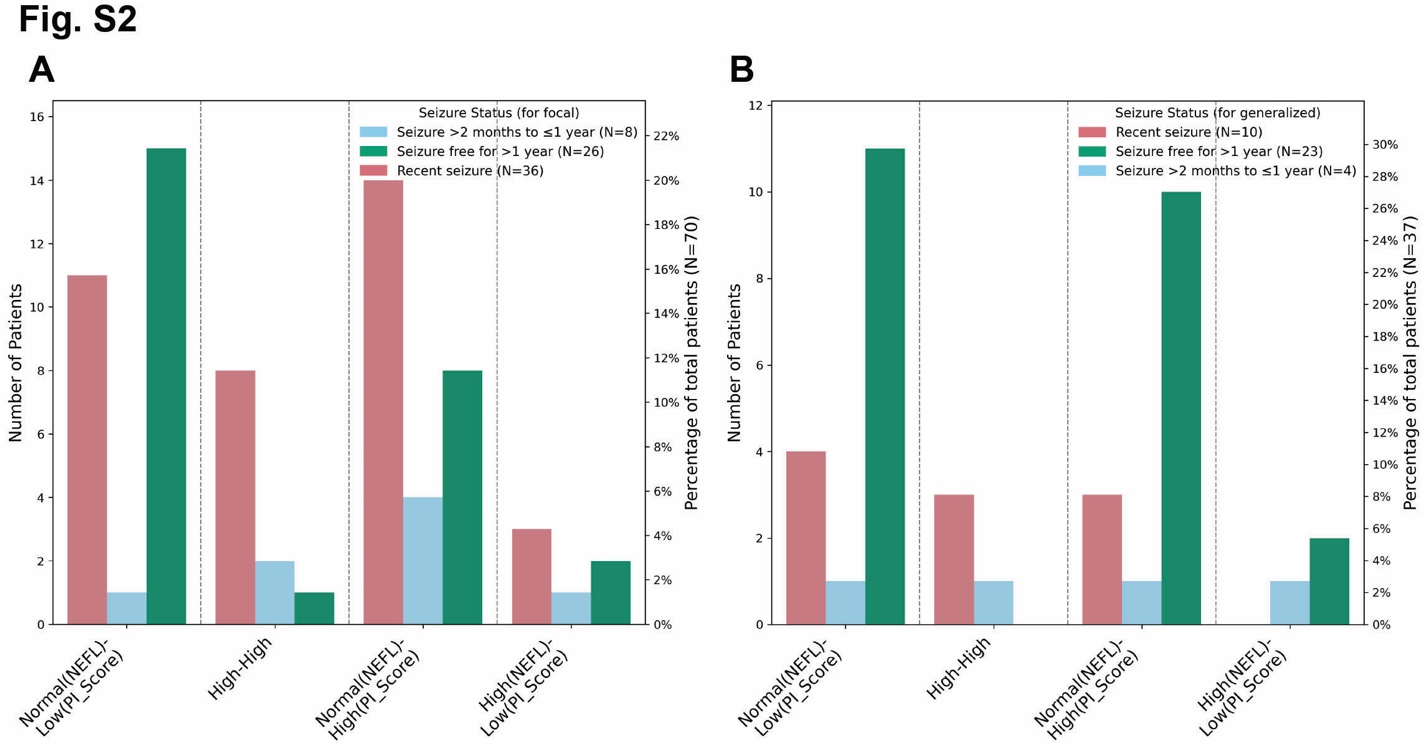


**Fig. S2: Seizures status analysis of focal and generalized patients.** Patients were grouped into four quadrants based on NEFL and PI_Score. (**A**) The bar plot shows patients with focal epilepsy in each quadrant (excluding the “unclassified”) according to their seizure status. (**B**) The bar plot shows patients with generalized epilepsy in each quadrant (excluding the “unclassified”) according to their seizure status.


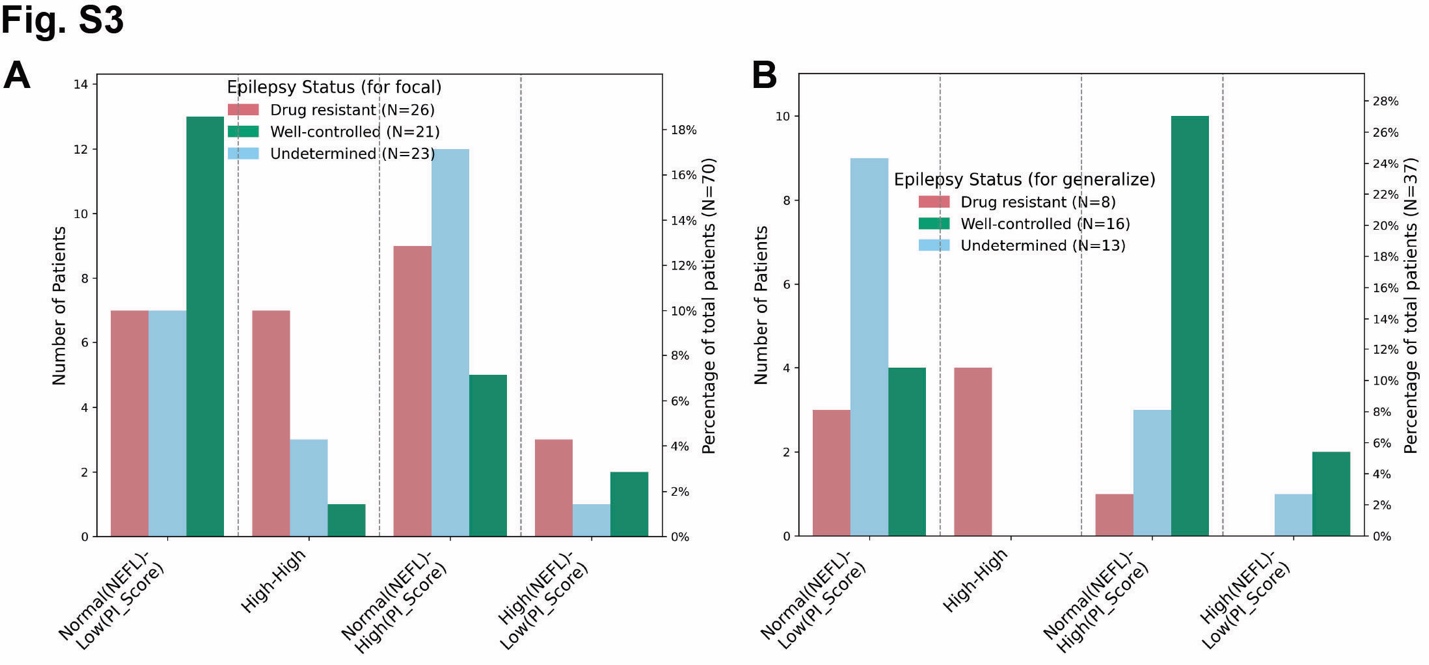


**Fig. S3: Epilepsy status analysis of focal and generalized patients.** Patients were grouped into four quadrants based on NEFL and PI_Score. (**A**) The bar plot shows patients with focal epilepsy in each quadrant (excluding the “unclassified”) according to their epilepsy status. (**B**) The bar plot shows patients with generalized epilepsy in each quadrant (excluding the “unclassified”) according to their epilepsy status.
